# Case Report: A case suffered acute cerebral infarction after removal of temporary cardiac pacing lead which led to the perforation of interventricular septum

**DOI:** 10.3389/fcvm.2024.1429480

**Published:** 2024-08-08

**Authors:** Lu Zhang, Jiechu Chen, Kailun Zhu, Jianghai Liu, Guiyang Li, Dong Chang, Qiang Li

**Affiliations:** ^1^ Department of Cardiology, Xiamen Cardiovascular Hospital, Xiamen University, Xiamen, China; ^2^ Julius-Maximilians-University Wuerzburg, Wuerzburg, Germany; ^3^ School of Medicine, Xiamen University, Xiamen, China

**Keywords:** cardiac pacing, artificial, anticoagulants, embolism and thrombosis, brain, septal perforation

## Abstract

We report an elderly male patient with frequent episodes of dizziness due to a complete atrioventricular block who underwent temporary pacemaker insertion in a local hospital. After the implantation of a permanent pacemaker and removal of the temporary pacemaker lead, the patient developed sudden neurological symptoms, upon which an acute cerebellar infarction was diagnosed via head CT. We will discuss the adequacy of the periprocedural administration.

## Introduction

A 71-year-old man was admitted to a local hospital because of intermittent dizziness 5 days ago. His electrocardiogram showed significant bradycardia and third-degree atrioventricular block with a ventricular rate of 29 beats per minute (BPM). The temporary pacemaker was administered for 3 days and the patient was transferred to our hospital for permanent pacemaker implantation.

**Figure 1:**
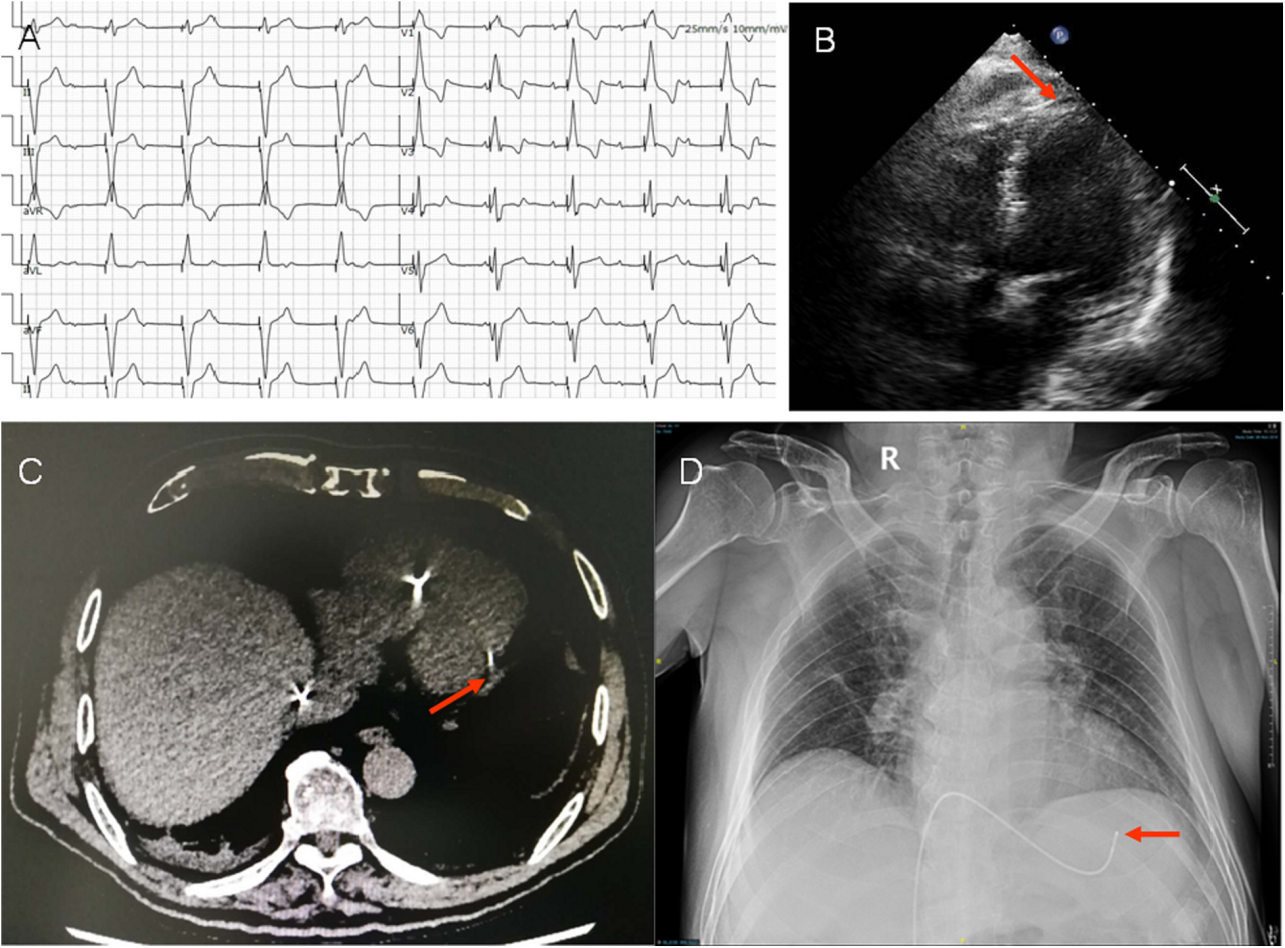
(**A**) Electrocardiogram (ECG) post temporary pacing. ECG showed *P* wave was not associated with QRS complex, indicated AV blocked. The axis of pacing QRS showed significant left deviation (−87^o^). The QRS pattern of V1 with a positive deflection and S wave in final part of V5-V6, showed the tip of temporary lead in the free wall of left ventricle. (**B**) Image showed the tip of the temporary lead located in the left ventricle with crossing the interventricular septum (arrow) in apical 4-chamber view. (**C**,**D**) The lead tip close to the left posterior free wall (arrow) was confirmed by lung CT and chest x-ray.

The patient was presented to us in good and stable physical condition, showing no signs of complication through the previous procedure. An electrocardiogram (ECG) showed a sinus rhythm (Figure 1A), third-degree atrio-ventricular block and left ventricular pacing pattern with complete right bundle branch block (RBBB). Blood examination showed an elevated D-dimer level at 3.93 mg/L (normal range 0–0.55 mg/L), and the fibrin degradation product was 8.43 µg/ml (normal range <5 µg/ml). No thrombosis was detected by ultrasound imaging in the lower extremities. Considering that the patient had been mostly immobilized for 3 days and was prone to thrombosis, enoxaparin sodium injection of 40 mg was administered subcutaneously every 12 h.

In the echocardiogram, we found the tip of the temporary pacemaker led to be located in the left ventricle with its lead crossing the interventricular septum and a small amount of circumferential pericardial effusion with no obvious perforation (Figure 1B). This misplacement was confirmed by lung CT and chest x-ray, showed the lead tip close to the left posterior free wall (Figures 1C,D).

**Figure 2:**
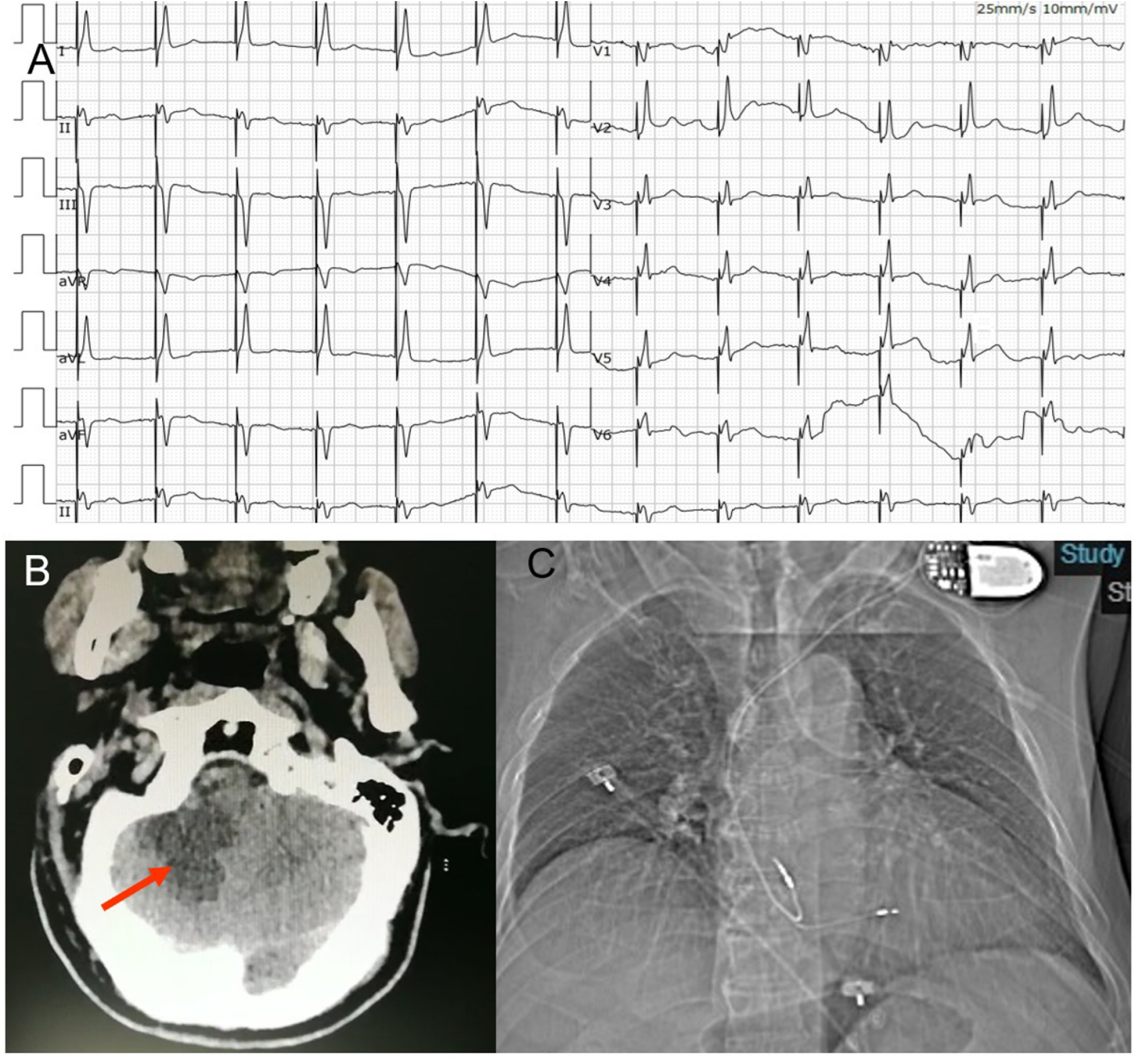
(**A**) ECG post permanent pacemaker implantation. The ECG suggested deep septal pacing with the tip of the lead close to the left posterior branch. (**B**) Craniocerebral CT- scan showed an infarction in his right cerebellum (arrow). (**C**) Anteroposterior chest radiograph of dual-chamber pacemaker implantation.

Next day, the placement of a dual-chamber pacemaker (Figure 2C) was carried out as planned, using the Select Secure pacing lead (Model 3830 69 cm, Medtronic Inc., Minneapolis, MN, USA) for left bundle branch area pacing. ECG monitoring system showed proper function of the permanent pacemaker (Model REDR01, Medtronic Inc., Minneapolis, MN, USA) and then the temporary pacing electrode was removed under the monitoring of x-ray; (Supplementary Videos S1, S2) and transthoracic echocardiography. During the procedure, we encountered no unexpected events and the patient's vital signs remained stable. ECG indicated left bundle branch pacing with incomplete RBBB pattern (Figure 2A). The amount of the pericardial effusion appeared to be unchanged. The patient had no subjective complaints.

On the second day, at 16 h after the procedure, the patient suddenly became developed unclear symptoms after forced defecation, including vomiting, vague speech, cold sweat, consciousness reduction and involuntary movements. His vital signs showed an elevated blood pressures at 150/89 mmHg. The neurological examination presented no cranial nerve dysfunction, symmetric deep-tendon reflexes, a negative Babinski sign and a slight weakness in all muscles. Coordination and sensory functions could not be tested. Upon this, a craniocerebral CT-scan was performed, showing an infarction in his right cerebellum (Figure 2B). The patient was transferred to the neurology department for further treatment.

## Discussion

We report a rare complication of cerebral infarction after removal of a temporary transvenous pacemaker lead. In our case, we confirmed that the temporary pacing lead has gone through the interventricular septum and even reached, albeit not yet perforated, the left ventricular free wall. One can assume that the during and after this procedure, ECG hasn't been properly evaluated, neither had the position of the lead been controlled through imaging. Unfortunately, the obvious changes of ECG were not recognized by local hospitals. Patients was transferred to our hospital 3 days later in order to further line of permanent pacemaker implantation. Usually, the right ventricular apex is the most common pacing site, and with left axis deviation, showing the pattern of left bundle branch block. The right ventricular apical pacing produces negative deflections in V_1_, instead of positive deflections (Figure 1A). The ECG of the case showed the tip of lead reached to the inferior, lateral wall of left ventricle. The patient with no history of cardiac structural disease or ischemic heart disease, and the cause of ventricular septal perforation was related to the improper operation of the operator.

It is unclear which factor led to the cerebellar infarction after the intervention. The high level of D-dimer before operation suggested that thrombosis might have pre-existed. After implantation of transvenous pacing, the venous circulation was affected in part, which caused decrease of circulation and a prothrombotic state (1). The indwelling sheath tube of temporary pacemaker may have led to thrombus formation. However, the likelihood of cerebral thromboembolism is rather low unless there is an anatomic basis for paradoxical embolism.

At this point, we would like to mention that thrombosis in the left ventricle had been ruled out through preoperative echocardiography and lung CT before permanent pacemaker implantation. However, these imaging methods have some limitations regarding the contrast of the obtainable images. Therefore, we should further improve the imaging data of the patient before the intervention, including left ventricular contrast echocardiography and cardiac magnetic resonance imaging, in order to detect potential thrombosis.

In addition, adequate anticoagulant therapy is essential. Some current studies have focused on the antithrombotic management of patients with temporary pacemakers, without reaching definitive consensus. One of these studies shows that temporary transvenous femoral pacemakers are associated with a significant risk of deep venous thrombosis (DVT), and conclude that adjunctive therapeutic anticoagulation (with heparin) is safe and effective in reducing the risk of DVT (2).

Although anticoagulation therapy had been introduced only once the patient was admitted to our hospital and that thrombotic status could have not been avoided by prophylactic therapy. Our patient in this case, who was treated with LMWH for 1.5 days, still suffered cerebral infraction after pacemaker lead removal. Therefore, in similar cases, usage, dosage and longevity of anticoagulant drugs are significant parameters which can be optimized before the intervention. Currently speaking, this issue still lacks evidence and consensus, which provides need for further exploration.

## Conclusion

Involuntary perforation of the interventricular septum during pacemaker installation is rather rare and can involve complications such as pericardial tamponade, further arrhythmias, embolic stroke etc. Unfortunately, no guideline yet exists regarding the periprocedural administration of cases like this. This provides even further need for careful individual evaluation of each patient which had undergone previous complicated procedures. As earlier mentioned, the optimization of the anticoagulative therapy could be essential to the prevention of similar incidents.

## Data Availability

The original contributions presented in the study are included in the article/Supplementary Material, further inquiries can be directed to the corresponding author.
